# Both Serum Apolipoprotein B and the Apolipoprotein B/Apolipoprotein A-I Ratio Are Associated with Carotid Intima-Media Thickness

**DOI:** 10.1371/journal.pone.0054628

**Published:** 2013-01-24

**Authors:** Fei Huang, Zhi Yang, Baihui Xu, Yufang Bi, Min Xu, Yu Xu, Jieli Lu, Yu Liu, Meng Dai, Wenzhong Zhou, Weiqing Wang, Yuhong Chen

**Affiliations:** 1 Key Laboratory for Endocrine and Metabolic Diseases of Ministry of Health, Rui-Jin Hospital, Shanghai Jiao Tong University School of Medicine, E-Institute of Shanghai Universities, Shanghai, China; 2 Shanghai Clinical Center for Endocrine and Metabolic Diseases, Shanghai Institute of Endocrine and Metabolic Diseases, Department of Endocrinology and Metabolism, Rui-Jin Hospital, Shanghai Jiao Tong University School of Medicine, Shanghai, China; University of Bari & Consorzio Mario Negri Sud, Italy

## Abstract

**Background:**

Previous studies indicated that apolipoprotein measurements predicted cardiovascular disease (CVD) risk; however, associations between apolipoproteins and carotid intima-media thickness (CIMT) were less explored.

**Methodology and Principal Findings:**

The cross-sectional study included 6069 participants aged 40 years or older with NGT from Shanghai, China. Serum fasting traditional lipids (total cholesterol [TC], low-density lipoprotein cholesterol [LDL-C], high-density lipoprotein cholesterol [HDL-C] and triglycerides [TG]), apoA-I and apoB were assessed. A high-resolution B-mode ultrasonography was performed to measure CIMT. We found CIMT increased progressively across the quartiles of serum apoB (p for trend <0.0001). In logistic regression, concentrations of apoB (odds ratio [OR] 1.27, 95% confidence interval [CI] 1.18–1.36), TC (OR 1.23, 95% CI 1.14–1.32), LDL-C (OR 1.25, 95% CI 1.16–1.34) and TG (OR 1.11, 95% CI 1.04–1.20) were significantly related to elevated CIMT after adjusted for age and sex. Meanwhile, the apoB/apoA-I ratio (OR 1.25, 95% CI 1.17–1.34) related to elevated CIMT. ApoB (OR 1.23, 95% CI 1.00–1.51) and the apoB/apoA-I ratio (OR 1.19, 95% CI 1.04–1.36) remained significantly associated with elevated CIMT, after adjusted for the traditional CVD risk factors including traditional lipids.

**Conclusions and Significance:**

There were significant associations between serum apoB, the apoB/apoA-I ratio and elevated CIMT. Serum apoB and the apoB/apoA-I ratio might be independent predictors of early atherosclerosis in NGT.

## Introduction

Dyscholesterolemia, including higher low-density lipoprotein cholesterol (LDL-C) and lower high-density lipoprotein cholesterol (HDL-C) is identified as independent risk factor for the development of cardiovascular disease (CVD) [1], [2]. LDL-C is widely used to assess the risk of vascular disease and the response to therapy. Current guidelines for coronary heart disease (CHD) prevention emphasize the use of total cholesterol (TC) and LDL-C in CHD risk assessment [1], [3]. However, there are accumulating evidences suggesting that apolipoproteins are better predictors for risk of CVD than traditional lipid parameters currently used in clinical practice. Several large, prospective epidemiological studies have shown that the apolipoprotein B (apoB) was a good predictor for the occurrence of CVD in the general population. In statin-treated patients with known CHD, apoB/apolipoprotein A-I (apoA-I) ratio, but not LDL-C/HDL-C ratio, was positively associated with clinical events [4]. John et al [5] thought that apoB should be added into the routine lipid panel for assessing and monitoring patients at risk for adverse outcomes.

Intima-media thickness of the carotid arteries is an early marker of atherosclerosis [6], [7], and it is also demonstrated as a risk factor for myocardial infarction and stroke in older adults [8]. In type 2 diabetics, compared with conventional lipids, the apoB/apoA-I ratio had a more closed association with carotid intima-media thickness (CIMT) [9]. Whether the same applies to subjects with normal glucose tolerance (NGT) is less well defined.

This present study was to assess whether the associations of apoB, apoA-I and the apoB/apoA-I ratio with CIMT were independent of traditional CVD risk factors and clinical routine lipid measurements in middle-aged and elderly Chinese with NGT.

## Methods

### Ethics Statement

The study protocol was approved by the Institutional Review Board of the Rui-jin Hospital affiliated to Shanghai Jiao-Tong University School of Medicine. Written informed consent was obtained from each participant before data collection.

### Study Design and Participants

We performed a cross-sectional health survey between March and August 2010, in Jiading district of Shanghai, China. Residents who aged 40 years and older were invited to participate by examination notice and home visits, during which we collected information on lifestyle, medical history and the use of medications using a standard questionnaire, and performed anthropometrical measurements. We used a simplified 75-g oral glucose tolerance test (OGTT) with fasting and 2-hour post-loading blood sampling to evaluate the glucose metabolism status. Totally there were 10375 subjects taken part in the survey. Based on the fasting plasma glucose (FPG) <6.1 mmol/L and 2 hour post-loading plasma glucose <7.8 mmol/L and without taking any anti-diabetic treatment, we enrolled 6117 subjects with NGT [10]. Among the enrolled participants, individuals with no information on CIMT (n = 48) were excluded, resulting in 6069 records eligible for the eventual analysis.

### Clinical and Biochemical Measurements

During the face-to-face interview, a standard questionnaire was administered by trained staff to collect information on health status, medications and lifestyle risk factors. The interview also included questions on history of chronic diseases, such as, the diagnosis and treatment of hypertension and diabetes. Current smokers or drinkers were subjects who smoked cigarettes or consumed alcohol regularly in the past 6 months.

Clinical examinations, including blood pressure and anthropometric measurements, were performed by trained clinical staff. Blood pressure was measured with an automated electronic device (OMRON Model HEM-752 FUZZY, Omron Company, Dalian, China) three times consecutively with 1 min intervals in a sitting position after 5-min rest. Blood pressure was the average of three measurements. Height and weight were recorded to the nearest 0.1 cm and 0.1 kg while participants were wearing light indoor clothing without shoes. Body mass index (BMI) was calculated as weight (kg) divided by squared height (meter). We measured waist circumference (WC) and hip circumference (HC) to the nearest 0.1 cm with participants in standing position.

After at least 10 hours of overnight fasting, venous blood samples were collected for measurements of serum lipid profile and plasma glucose. The fasting time was verified before the blood specimen was taken. Fasting serum insulin concentrations were determined by an electrochemiluminescence assay (Roche -Diagnostics, Switzerland). The homeostasis model assessment of insulin resistance (HOMA-IR) was calculated according to the equation described by Matthews et al [11]. FPG and serum TC, triglycerides (TG), HDL-C, LDL-C, apoB and apoA-I were measured using an automated biochemical instrument (Bayer Biochemical autoanalyzer ADVIA 1650, Leverkusen, Germany). The coefficient of variation (%) was 1.41 for TG, 1.40 for TC, 1.42 for LDL-C, 2.99 for HDL-C, 1.29 for apoB, 1.49 for apoA-1.

### CIMT Measurements

CIMT measurements were performed by an experienced sonographer using a high-resolution B-mode tomographic ultrasound system (Phillips IU22, Japan) with a linear 7.5- to 10-MHz transducer. The operator measured CIMT on the far wall of the right and left common carotid arteries, 1.5 cm proximal to the bifurcation. The transducer was manipulated so that the lumen diameter was maximized in the longitudinal plane. CIMT was measured on-line at the end of diastole as the distance from the leading edge of the first echogenic line to that of the second echogenic line. The first and second lines represented the lumen–intimal interface and the collage-contained upper layer of tunic adventitia, respectively. The greater value of the right or left common CIMT was used for analysis. Values above the upper tenth of CIMT (≥0.7 mm [12]) were defined as elevated CIMT.

### Statistical Analyses

All statistical analyses were performed on SAS version 9.2 (SAS Institute Inc, Cary, NC). The characteristics of the study population were described according to quartiles of apoB. Values were presented as means ± S.D. or geometrical means (95% confidence interval [CI]) for continuous variables and percentages for categorical variables. Fasting serum insulin, HOMA-IR and triglycerides were analyzed after log-transformation due to a skewed distribution. Pearson’s correlation coefficients were used to measure linear associations between the different risk factors and CIMT. Stepwise regression analysis was used to identify independent determinants of CIMT. The unadjusted and multivariate adjusted logistic regression analysis was used to explore the associations of serum apoB concentrations and the apoB/apoA-I ratio with elevated CIMT compared with conventional lipids and other risk factors. The associations of apoB and the apoB/apoA-I ratio with elevated CIMT were further explored by categorizing apoB and the apoB/apoA-I into quartiles and using the first quartile as the reference. Adjusted odds ratios (ORs) and corresponding 95% CIs were estimated with the use of multivariate logistic regression analysis models.

A p value <0.05 was considered statistically significant.

## Results

### Characteristics of the Study Population

General demographic and laboratory characteristics of the 6069 participants are shown in Table 1. The median age of the population was 56.3 years old, and the proportion of men was 38.2%. The median concentration of apoB was 0.94 g/L. Compared with the first quartile, participants in the highest quartile were older and had a lower proportion of men. Across apoB quartiles, the levels of BMI, WC, HC, systolic blood pressure (SBP), diastolic blood pressure (DBP), lipids (TG, TC, LDL-C), FPG, fasting serum insulin, HOMA-IR, apoA-I, and the apoB/apoA-I ratio were gradually and significantly increased, whereas the concentrations of HDL-C were decreased. CIMT level was also increased in participants with higher apoB level. After adjusting for age, sex, the increase of CIMT according to the apoB quartiles were not substantially changed (p for trend <0.0001).

**Table 1:** Characteristics of study population.

	Median (Interquartile Range)/Number (%)	ApoB quartiles (g/L)				p fortrend^a^	p fortrend^b^
		Quartile 1	Quartile 2	Quartile 3	Quartile 4		
		(≤0.78)	(0.78–0.92)	(0.92–1.08)	(>1.08)		
Participants, n	6069	1458	1488	1550	1573	–	–
ApoB, g/L	0.94±0.23	0.68±0.07	0.85±0.04	0.99±0.04	1.24±0.16	–	–
Age, yr	56.3±9.5	55.8±10.1	56.6±9.8	57.4±9.2	58.1±8.7	<0.0001	–
Male, n (%)	2321 (38.2)	624 (42.8)	619 (41.6)	536 (34.6)	542 (34.5)	<0.0001	–
Current smoker, n (%)	1347 (22.9)	354 (25.1)	359 (25.0)	299 (19.9)	334 (21.9)	0.0015	0.28
Current drinker, n (%)	605 (10.3)	138 (9.8)	167 (11.5)	134 (8.9)	166 (10.9)	0.41	0.0072
BMI, kg/m^2^	24.1±3.1	23.7±3.1	24.4±3.0	24.9±3.1	25.2±3.0	<0.0001	<0.0001
WC, cm	81.0±8.7	78.2±8.8	80.6±8.4	82.0±8.4	83.2±8.3	<0.0001	<0.0001
HC, cm	93.5±5.6	92.1±5.7	93.3±5.4	94.0±5.5	94.4±5.5	<0.0001	<0.0001
SBP, mm Hg	137±19	134±19	135±19	138±19	140±19	<0.0001	<0.0001
DBP, mm Hg	82±10	80±10	81±10	82±10	83±10	<0.0001	<0.0001
TG, mmol/L	1.24 (0.90–1.75)	0.93 (0.72–1.29)	1.12 (0.85–1.54)	1.33 (1.02–1.84)	1.58 (1.22–2.11)	<0.0001	<0.0001
TC, mmol/L	5.23±0.94	4.31±0.58	4.91±0.55	5.40±0.54	6.23±0.81	<0.0001	<0.0001
HDL-C, mmol/L	1.35±0.32	1.38±0.33	1.36±0.34	1.34±0.32	1.33±0.29	<0.0001	<0.0001
LDL-C, mmol/L	3.12±0.83	2.23±0.41	2.82±0.35	3.27±0.38	4.07±0.69	<0.0001	<0.0001
FPG, mmol/L	4.97±0.46	4.87±0.46	4.96±0.46	4.97±0.46	5.05±0.45	<0.0001	<0.0001
Fasting serum insulin, µIU/mL	6.2 (4.2–8.7)	5.1 (3.3–7.4)	5.9 (4.1–8.2)	6.6 (4.5–9.1)	7.0 (5.2–9.6)	<0.0001	<0.0001
HOMA-IR	1.36 (0.92–1.94)	1.10 (0.70–1.59)	1.31 (0.89–1.81)	1.44 (0.97–2.02)	1.57 (1.14–2.17)	<0.0001	<0.0001
ApoA-I, g/L	1.26±0.27	1.22±0.28	1.24±0.26	1.27±0.28	1.28±0.26	<0.0001	<0.0001
ApoB/apoA-I ratio	0.78±0.24	0.58±0.15	0.71±0.15	0.81±0.18	1.00±0.23	<0.0001	<0.0001
CIMT, mm	0.57±0.10	0.55±0.10	0.57±0.10	0.58±0.11	0.59±0.11	<0.0001	<0.0001

Data are means ± S.D. or medians (interquartile ranges) for skewed variables, or numbers (proportions) for categorical variables. Linear or logistic regression is applied to analyze trend differences across apoB groups. Skewed variables are logarithmically transformed before analysis.

a Unadjusted.

b Adjusted for age, sex.

Abbreviations: ApoB, apolipoprotein B; BMI, body mass index; WC, waist circumference; HC, hip circumference; HOMA-IR, homeostasis model assessment of insulin resistance; SBP, systolic blood pressure; DBP, diastolic blood pressure; TG, triglycerides; TC, total cholesterol; HDL-C, high-density lipoprotein cholesterol; LDL-C, low-density lipoprotein cholesterol; FPG, fasting plasma glucose; ApoA-I, apolipoprotein A-I; CIMT, carotid intima-media thickness.

### Prevalence of Elevated CIMT in Different apoB Levels

**Figure 1:**
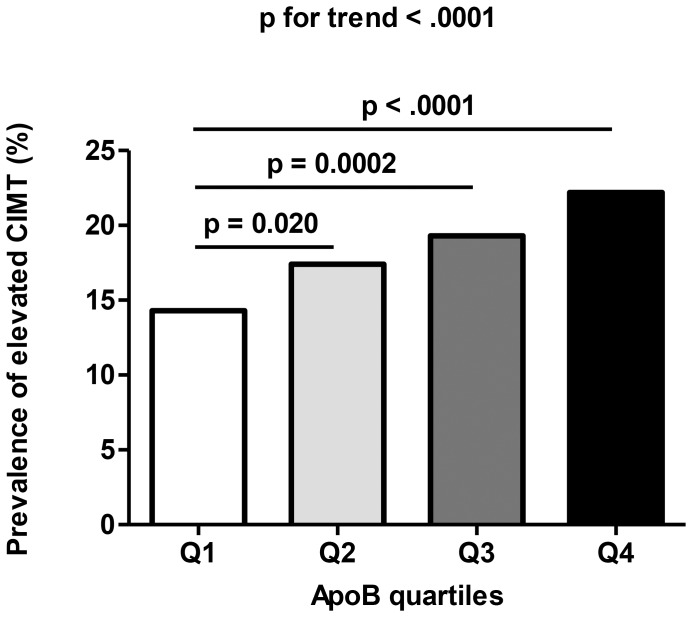
Prevalence of elevated CIMT in different quartiles of apoB: quartile 1 (Q1, n = 167), 0–0.78 g/L; quartile 2 (Q2, n = 216), 0.78–0.92 g/L; quartile 3 (Q3, n = 257), 0.92–1.08 g/L; quartile 4 (Q4, n = 298), >1.08 g/L.

Figure 1 illustrated that the prevalence of elevated CIMT differed statistically in different apoB groups. Across the apoB quartiles, the prevalence of elevated CIMT were 14.3, 17.4, 19.3 and 22.2%, respectively (p for trend <0.0001, Figure 1.). Compared with the lowest quartile, the prevalence of elevated CIMT of the second, third and fourth quartile of serum apoB were significant higher (p = 0.020, 0.0002 and <0.0001, respectively).

### Correlations of Serum apoB and the apoB/apoA-I ratio with CIMT

Pearson correlation analyses revealed that age, sex, smoking status, drinking status, BMI, WC, HC,SBP, DBP, FPG, fasting serum insulin, HOMA-IR, TC, TG, LDL-C, HDL-C, apoB and the apoB/apoA-I ratio were significantly related with CIMT (Table 2). Multivariate stepwise linear regression analyses revealed that apart from traditional and well-recognized risk factors of CIMT such as age, sex, BMI, SBP, DBP, fasting serum insulin, HOMA-IR and TC, apoB was also an independent determinant of CIMT; however, no association was observed between the apoB/apoA-I ratio and CIMT in the multivariate analyses (Table 2).

**Table 2:** Pearson’s correlation and multiple stepwise linear regression analysis of risk factors associated with CIMT.

	r	p value	*β* ± SE	p value
Age (yr)	0.48	<0.0001	0.0044±0.00015	<0.0001
Sex (male = 1, female = 0)	0.26	<0.0001	0.042±0.0033	<0.0001
Current smoker (yes = 1, no = 0)	0.15	<0.0001	0.012±0.0037	0.0010
Current drinker (yes = 1, no = 0)	0.10	<0.0001		
BMI (kg/m^2^)	0.13	<0.0001	0.0032±0.0004	<0.0001
WC (cm)	0.18	<0.0001		
HC (cm)	0.05	<0.0001		
SBP (mm Hg)	0.27	<0.0001	0.00078±0.00009	<0.0001
DBP (mm Hg)	0.038	0.0030	−0.00098±0.00017	<0.0001
FPG (mmol/L)	0.059	<0.0001		
Log fasting serum insulin (µIU/mL)	−0.073	<0.0001	−0.017±0.0039	<0.0001
Log HOMA-IR	−0.064	<0.0001		
TC (mmol/L)	0.081	<0.0001	−0.005±0.0023	0.028
Log TG (mmol/L)	0.051	<0.0001		
LDL-C (mmol/L)	0.10	<0.0001		
HDL-C (mmol/L)	−0.053	<0.0001		
ApoA-I (g/L)	−0.0064	0.62		
ApoB (g/L)	0.13	<0.0001	0.065±0.0096	<0.0001
ApoB/apoA-I ratio	0.11	<0.0001		

### Comparisons of Elevated CIMT in Relations to Apolipoproteins and Clinical Routine Lipids

Serum TC, LDL-C, apoB and the apoB/apoA-I ratio were significantly related to elevated CIMT when adjusted for traditional CVD risk factors, including age, sex, BMI, WC, HC, medical treatment, smoking habits, drinking habits, SBP, DBP, FPG, fasting serum insulin and HOMA-IR (Model 2, Table 3). After further adjustment for the four traditional lipids – namely, TC, Log TG, LDL-C and HDL-C based on Model 2, apoB and the apoB/apoA-I ratio, remained significantly associated with elevated CIMT (OR 1.23, 95% CI 1.00–1.51, p = 0.049; OR 1.19, 95% CI 1.04–1.36, p = 0.014; respectively). No significant associations were observed between TC (OR 1.04, 95% CI 0.89–1.21), LDL-C (OR 1.03, 95% CI 0.86–1.24), Log TG (OR 0.99, 95% CI 0.91–1.08) and elevated CIMT, after further adjustment for apoB and apoA-I based on Model 2 (Table 3).

**Table 3:** Odds ratios per standard deviation increment of different lipids or lipid ratio with a 95% confidence interval for elevated CIMT.

	Model 1	p value	Model 2	p value	Model 3**	p value
TC	1.23 (1.14–1.32)	<0.0001	1.21 (1.12–1.31)	<0.0001	1.04 (0.89–1.21)	0.62
HDL-C	0.95 (0.88–1.03)	0.21	0.98 (0.90–1.07)	0.69	1.06 (0.94–1.19)	0.33
LDL-C	1.25 (1.16–1.34)	<0.0001	1.25 (1.15–1.35)	<0.0001	1.03 (0.86–1.24)	0.72
Log TG	1.11 (1.04–1.20)	0.0038	1.07 (0.99–1.16)	0.11	0.99 (0.91–1.08)	0.88
ApoB	1.27 (1.18–1.36)	<0.0001	1.27 (1.17–1.37)	<0.0001	1.23 (1.00–1.51)	0.049
ApoA-I	0.94 (0.87–1.01)	0.099	0.96 (0.89–1.04)	0.35	0.93 (0.83–1.04)	0.22
ApoB/apoA-I ratio	1.25 (1.17–1.34)	<0.0001	1.24 (1.15–1.34)	<0.0001	1.19 (1.04–1.36)	0.014

Data are odds ratios (ORs, 95% confidential intervals).

Model 1is adjusted for age and sex;

Model 2 is adjusted for age, sex, BMI, WC, HC, medical treatment, smoking habits, drinking habits, SBP, DBP, FPG, Log fasting serum insulin and Log HOMA-IR;

Model 3**, for the associations with TC, HDL-C, LDL-C and Log TG, respectively, the adjustment included ApoB, and ApoA-I based on Model 2; For the associations with ApoB, ApoA-I and ApoB/apoA-I ratio, respectively, the adjustment included TC, HDL-C, LDL-C and Log TG based on Model 2.

The category analysis for serum apoB concentrations associated with elevated CIMT was confirmatory. As compared to quartile 1, quartile 2 (OR 1.29, 95% CI 1.00–1.66), 3 (OR 1.47, 95% CI 1.09–1.96) or 4 (OR 1.59, 95% CI 1.07–2.35) of apoB concentration were significantly associated with elevated CIMT, respectively, after the full adjustments (p for trend = 0.017, Table 4). Broadly similar associations were noted for the apoB/apoA-I ratio, with adjusted elevated CIMT ORs for increasing quartiles of the apoB/apoA-I ratio of 1.00 (reference), 1.50 (95% CI, 1.17–1.92), 1.52 (95% CI, 1.14–2.03) and 1.43 (95% CI, 1.00–2.05) (p for trend = 0.12, Table 4).

**Table 4:** The risk of elevated CIMT according to quartiles of serum apoB and the apoB/apoA-I ratio.

	Model 1	Model 2	Model 3
ApoB (g/L)			
Quartile 1 (≤0.78)	1.00 (reference)	1.00 (reference)	1.00 (reference)
Quartile 2 (0.78–0.92)	1.34 (1.07–1.67)	1.38 (1.09–1.74)	1.29 (1.00–1.66)
Quartile 3 (0.92–1.08)	1.65 (1.33–2.06)	1.66 (1.32–2.09)	1.47 (1.10–1.97)
Quartile 4 (>1.08)	1.94 (1.57–2.40)	1.98 (1.57–2.50)	1.60 (1.08–2.36)
p for trend	<0.0001	<0.0001	0.015
ApoB/apoA-I ratio			
Quartile 1 (≤0.62)	1.00 (reference)	1.00 (reference)	1.00 (reference)
Quartile 2 (0.62–0.76)	1.55 (1.260–1.93)	1.61 (1.28–2.02)	1.51 (1.17–1.93)
Quartile 3 (0.76–0.92)	1.72 (1.39–2.14)	1.74 (1.38–2.20)	1.54 (1.15–2.05)
Quartile 4 (>0.92)	1.83 (1.48–2.26)	1.81 (1.43–2.30)	1.44 (1.01–2.06)
p for trend	<0.0001	<0.0001	0.11

Data are odds ratios (ORs, 95% confidential intervals).

Model 1 is adjusted for age and sex;

Model 2 is adjusted for age, sex, BMI, WC, HC, medical treatment, smoking habits, drinking habits, SBP, DBP, FPG, Log fasting serum insulin and Log HOMA-IR;

Model 3 is adjusted for age, sex, BMI, WC, HC, medical treatment, smoking habits, drinking habits, SBP, DBP, FPG, Log fasting serum insulin, Log HOMA-IR, TC, HDL-C, LDL-C and Log TG.

## Discussion

In the present study of 6069 community-dwelling Chinese participants aged 40 years or older with NGT, we found that serum apoB and the apoB/apoA-I ratio were strongly and positively associated with CIMT independently of traditional serum lipids and other conventional risk factors. To some extent, the results might suggest that the measurements of apolipoproteins were stronger predictors in carotid atherosclerosis than routine lipids.

Apolipoproteins are the protein components of plasma lipoproteins [13]. Several large prospective studies have showed that apoB is superior to TC or LDL-C to predict the risk of vascular disease [13]–[17]. In the Apolipoprotein-related Mortality Risk Study (AMORIS) [14], apoB was found to be a stronger marker of cardiovascular disease risk than LDL-C at any LDL-C levels, but especially in those having normal/low LDL-C levels. Moss et al. [15] investigated that apoB was significantly associated with increased coronary event rates, whereas LDL-C was not. Similar findings have been found in the Quebec Cardiovascular Study [16], the MONICA/KORA Augsburg cohort study [17] and the multi-ethnic US population study [18]. In the Cardiovascular Risk in Young Finns Study [19], apoB and apoA-I measured in children and adolescents reflected a lipoprotein profile predisposing to the development of subclinical atherosclerosis in adulthood.

Early detection and intervention of the risk factors of CVD, is of great importance for CVD. Of all the indicators of CVD, CIMT is an early marker and is most widely adopted [20], [21]. However, studies evaluating the associations between apolipoproteins and CIMT are limited. We demonstrated that both serum apoB concentrations and the apoB/apoA-I ratio were independently associated with elevated CIMT in middle aged and elderly Chinese with NGT. Furthermore, these associations remained even after adjustment for the conventional risk factors including traditional lipids. This observation indicated that apoB and the apoB/apoA-I ratio gave additional information in predicting risk for atherosclerotic disease, beyond that of LDL-C.

In our study, we also found that the increasing levels of apoB were associated with increased BMI, WC, SBP, DBP, TG, TC, LDL-C, FPG and fasting serum insulin. In the insulin resistance atherosclerosis study (IRAS), it also showed that serum apoB correlated positively to BMI, WC, DBP and fasting serum insulin [22]. Wallenfeldt et al [23] found that the metabolic syndrome patients had an increased production of small dense LDL particles, which had been shown to correlate with plasma apoB and considerable evidence indicated that these particles were atherogenic and thus, might have effect on the arterial wall.

The pathophysiological basis for why apolipoproteins are better than other conventional lipids is not completely established. According to the Response-to-Retention hypothesis of atherosclerosis, the key initiating process in atherosclerosis is the subendothelial retention of apoB-containing lipoprotein [24]. Biological responses to the retained lipoproteins, including a chronic and maladaptive macrophage- and T-cell–dominated inflammatory response, promote subsequent lesion development. The National Cholesterol Education Program (NCEP) Adult Treatment Panel (ATP) III [1] recommended initiation of therapy and therapeutic goals within 3 different CHD risk categories based on LDL-C levels. However, LDL particles, which contain most of the cholesterol in plasma, may differ in composition. Small dense LDL species are more atherogenic than larger LDL particles but carry less LDL-C [25]. Moreover, St-Pierre AC et al found that increased plasma CRP levels further elevate the risk of CHD associated with having small, dense LDL particles [26]. LDL-C therefore is not always equivalent to LDL particle number. By contrast, each VLDL and LDL particle contains 1 molecule of apoB; therefore, plasma apoB is equivalent to the total atherogenic particle number, more than 90% of which are LDL particles. In addition, van Deventer et al [27] found that direct LDL-C measurements did not improve the accuracy of CVD risk score classification.

Dysglycemia Current guidelines for CHD prevention emphasize the use of TC and LDL-C in CHD risk assessment [1], [3]. However, there has been an ongoing debate on whether apoB, or rather the apoB/apoA-I ratio, would be a more appropriate measure for estimating CHD risk associated with dyslipidaemia. A series of large, prospective epidemiological studies [28], [29] has recently shown that apoB is superior to LDL-C as a predictor of the risk of vascular disease. In a cross-sectional analysis of the US population [28], LDL-C was not significantly correlated with history of atherosclerotic disease. Similarly, in the recently published data from the Framingham study [29], LDL-C was also not found to be a significant risk factor of CHD, which suggested that LDL-C was not the best target for lipid-lowering treatment strategies. Meanwhile, apoB was first suggested as an alternative target treatment by the Canadian Working Group [30]. At present, the American Diabetes Association and the American College of Cardiology [31] have stated that apoB is the test of choice to assess the adequacy of statin treatment and should therefore be introduced into routine clinical practice. Furthermore, the methods can easily be automated and analyses are cheap, it can be performed on previously frozen sera, and importantly, non-fasted samples can be used.

Some limitations of our present study were of concern. First, the cross-sectional design of this study doesn’t provide information as to whether apolipoproteins predict the progression of CIMT, and cannot compare the associations of the different tests with clinical events from CVD. Second, the population included the subjects with dislipidemia, they might have been treated, but we made adjustment for the drug using history to control the bias. Third, we only used one test for apoB and the various apoB assays such as apoB-48 and apoB-100 may not show the same performance, however, apoB measurement was more convenient in laboratory. Finally, because most participants were residents of a rural community from shanghai, there was the possibility of selection bias. Although our study had limitations in generalizing its results to the worldwide population, the present study was meaningful as a first study to clarify the relationship between lipid and lipoprotein profiles and CIMT among an Asian population with NGT.

In conclusion, the present study showed that apoB and the apoB/apoA-I ratio were associated with elevated CIMT in middle-aged and elderly Chinese with NGT. In addition, the associations were independent of conventional lipids and other risk factors. Although more studies are needed to confirm these findings and to elucidate the mechanisms for this associations, our finds support the fact that apolipoprotein measurements may be better biomarkers of atherosclerotic disease than traditional lipids measures.

## Supporting Information

Table S1 **Odds ratios per standard deviation increment of different lipids or lipid ratio with a 95% confidence interval for elevated CIMT in metabolic syndrome population.** Data are odds ratios (ORs, 95% confidential intervals). Model 1 is adjusted for age and sex; Model 2 is adjusted for age, sex, BMI, WC, HC, medical treatment, smoking habits, drinking habits, SBP, DBP, FPG, Log fasting serum insulin and Log HOMA-IR; Model 3**, for the associations with TC, HDL-C, LDL-C and Log TG, respectively, the adjustment included ApoB, and ApoA-I based on Model 2; For the associations with ApoB, ApoA-I and ApoB/apoA-I ratio, respectively, the adjustment included TC, HDL-C, LDL-C and Log TG based on Model 2.
(DOC)
