# Supplementary material for: Both Serum Apolipoprotein B and the Apolipoprotein B/Apolipoprotein A-I Ratio Are Associated with Carotid Intima-Media Thickness
Source: PLoS One. 2013 Jan 24;8(1):e54628. doi: 10.1371/journal.pone.0054628 (PMC3554742; doi:10.1371/journal.pone.0054628)
Supplement: Table S1 — Odds ratios per standard deviation increment of different lipids or lipid ratio with a 95% confidence interval for elevated CIMT in metabolic syndrome population. Data are odds ratios (ORs, 95% confidential intervals). Model 1 is adjusted for age and sex; Model 2 is adjusted for age, sex, BMI, WC, HC, medical treatment, smoking habits, drinking habits, SBP, DBP, FPG, Log fasting serum insulin and Log HOMA-IR; Model 3**, for the associations with TC, HDL-C, LDL-C and Log TG, respectively, the adjustment included ApoB, and ApoA-I based on Model 2; For the associations with ApoB, ApoA-I and ApoB/apoA-I ratio, respectively, the adjustment included TC, HDL-C, LDL-C and Log TG based on Model 2. (DOC) [file pone.0054628.s001.doc]

**Table S1.** Odds ratios per standard deviation increment of different lipids or lipid ratio with a 95% confidence interval for elevated CIMT

|  | Model 1 | p value | Model 2 | p value | Model 3** | p value |
| --- | --- | --- | --- | --- | --- | --- |
| TC | 1.31 (1.22-1.41) | < 0.0001 | 1.28 (1.18-1.38) | < 0.0001 | 1.18 (1.00-1.38) | 0.051 |
| HDL-C | 1.02 (0.95-1.10) | 0.58 | 1.01 (0.93-1.09) | 0.89 | 1.12 (1.00-1.26) | 0.055 |
| LDL-C | 1.31 (1.22-1.41) | < 0.0001 | 1.29 (1.20-1.40) | < 0.0001 | 1.11 (0.91-1.35) | 0.32 |
| Log TG | 1.06 (0.99-1.14) | 0.10 | 1.09 (1.01-1.17) | 0.031 | 1.01 (0.92-1.10) | 0.89 |
| ApoB | 1.29 (1.20-1.39) | < 0.0001 | 1.29 (1.20-1.40) | < 0.0001 | 1.17 (0.96-1.44) | 0.13 |
| ApoA-I | 0.96 (0.89-1.03) | 0.26 | 0.96 (0.89-1.04) | 0.32 | 0.89 (0.80-1.00) | 0.042 |
| ApoB/apoA-I ratio | 1.28 (1.20-1.37) | < 0.0001 | 1.27 (1.18-1.37) | < 0.0001 | 1.26 (1.10-1.44) | 0.0011 |

Data are odds ratios (ORs, 95% confidential intervals).

Model 1is adjusted for age and sex;

Model 2 is adjusted for age, sex, BMI, WC, HC, medical treatment, smoking habits, drinking habits, SBP, DBP, FPG, Log fasting serum insulin and Log HOMA-IR;

Model 3**, for the associations with TC, HDL-C, LDL-C and Log TG, respectively, the adjustment included ApoB, and ApoA-I based on Model 2; For the associations with ApoB, ApoA-I and ApoB/apoA-I ratio, respectively, the adjustment included TC, HDL-C, LDL-C and Log TG based on Model 2.
